# Seasonal Herbage–Livestock Balance and Grassland Pressure Index Analysis in the Yellow River Source Park, Tibet Plateau, China

**DOI:** 10.1002/ece3.70586

**Published:** 2024-11-28

**Authors:** Yalin Wang, Yuanyue Geng, Xungang Wang, Tianwei Xu, Qian Zhang, Hongjin Liu, Na Zhao, Shixiao Xu

**Affiliations:** ^1^ Northwest Institute of Plateau Biology Chinese Academy of Sciences Xining China; ^2^ University of Chinese Academy of Sciences Beijing China

**Keywords:** annual carrying capacity, digestible crude protein, grassland pressure index, metabolic energy, nutritional output, seasonal carrying capacity

## Abstract

Grassland carrying capacity is an indicator for measuring the stability of grassland ecosystems and can provide a basis for formulating regional sustainable grazing strategies. However, most previous studies on this have only considered annual fluctuations, but seasonal changes were ignored. In this study, the herbage yield and nutrient value of two grassland types in Yellow River Source Park (YRSP) were measured by sampling point survey method in four seasons, and the seasonal and annual grassland carrying capacity, carrying numbers of standard sheep unit (SU) were estimated based on the dry matter (DM) content, crude protein, and metabolic energy of herbage. Due to the carrying capacity being low during the yellow and wintering periods, we combined them and calculated the carrying capacity for only three periods, including the flourishing period, greening period, and withering period. The grassland pressure index (GPI) was measured by the ratio of the actual standard sheep number and the calculated number. The results showed that the herbage yield and nutrient output were higher in spring and summer, lower in autumn and winter, and showed a tendency for alpine meadows to be higher than alpine grasslands during the flourishing period (*p* < 0.05). The unit area carrying capacity varied significantly with the season and showed the seasonal changing characteristics of the flourishing period > greening period > withering period. The seasonal carrying number was much higher than the actual carrying number during the flourishing period and much lower than the actual carrying number during the withering period. In terms of annual carrying capacity, the GPI was balanced when considering livestock alone, while critical overloaded when considering livestock and wildlife. This study suggested the influence of seasonal change on the grassland carrying capacity should be fully considered in the grassland utilization. Meanwhile, the feeding needs of livestock and wildlife should be taken into account, and timely supplemented when forage is in short supply.

## Graphical abstract

Seasonal disparity and imbalance of grassland carrying capacity and forage nutrient output are quantified. Annual carrying pressure was balanced when considering livestock alone, and critical overloaded when considering livestock and wildlife. Sustainable management is recommended with seasonally supplementary feeding and livestock population control.

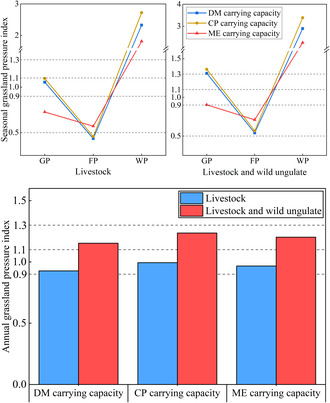

## 1 Introduction

Grassland ecosystems, comprised about 40% of Earth's surface, are important providers of livestock husbandry and contribute to the livelihoods of around 800 million people worldwide (Blair, Nippert, and Briggs 2014). Driven by population and income growth, livestock products already account for 17% of the total global energy intake and still increasing (Herrero et al. 2009). This has led to increased grazing pressure and excessive loss of carbon and biodiversity on grasslands, despite some livestock reducing their dependence through the use of crop‐based diets, genetic improvement, and the addition of preventive and growth‐promoting feed additives (Herrero et al. 2013).

Heavy stocking densities and overgrazing were important causes of grassland degradation, whereas properly managed grazing can contribute to avoid high stocking pressure (Cao et al. 2019; Yang, Zhao, and Chen 2022; Zhang et al. 2014). The benefits of moderate grazing have been widely proven to improve the functioning of grassland ecosystems, regulate the terrestrial carbon cycle, and increase ecological resilience to natural disasters (Du et al. 2020; Li et al. 2021; Zhang et al. 2023). This indicates that for sustainable grazing management, it is essential to determine an appropriate grazing intensity and number of grazing livestock on grasslands to optimize grassland utilization and meet the nutritional requirements of livestock (Umuhoza et al. 2021; C. Yang et al. 2018; F. Yang et al. 2018).

The grassland carrying capacity, a longstanding discussion in the literature, is used to assess stocking rates and is interpreted as the number of herbivores that can be supported per unit area of grassland while ensuring the ecological, economic, and social needs for sustainable development (Chapman and Byron 2018; Freeland and Choquenot 1990; Meshesha, Moahmmed, and Yosuf 2019). The estimation of grassland carrying capacity is typically based on pasture yield, and the yield acquisition method commonly involves direct harvesting, yield simulation models, and remote sensing model measurements (Umuhoza et al. 2021; Xu et al. 2008; F. Yang et al. 2018). For instance, Piipponen et al. used MODIS Net Primary Production product to estimate the aboveground biomass (AGB) and then to calculate livestock carrying capacities at a global level (Piipponen et al. 2022). Cai et al. (2022) used remote sensing to estimate grassland productivity and calculated the carrying capacity of grassland in terms of both wildlife and domestic animals. Recent studies have recommended that plant nutrition status and livestock nutritional demands be taken into account when calculating grassland carrying capacity, as livestock carrying capacity based on edible pasture alone does not reveal the exact carrying potential of grasslands and it is difficult to assess and provide for the nutrient requirements of herbivores (Xu, Quan, et al. 2020; Xu, Wang, et al. 2020; C. Yang et al. 2018; Zha et al. 2022). In addition, the different estimations were often inconsistent, and the lower estimate should be considered as the reference for the sustainable utilization and protection of grassland (Yu et al. 2021; Sun et al. 2015; Xu, Quan, et al. 2020; Xu, Wang, et al. 2020).

However, these studies have limited research on seasonal changes in carrying capacity. In one of the few articles on seasonal grazing intensity, Fetzel et al. (2017) estimated global seasonal limitations to grazing intensity by combining monthly net primary production data and a map of global livestock distribution and suggested that 39% of the total global natural grasslands lie below their potential. Although the calculations included grasslands around the world, evaluation of the available forage is still constrained by regional climate, topography, and grassland type. Thus, the assessment of the carrying capacity of grassland at the regional level was necessary to maintain the productivity of both animals and forage and sustain the health of grassland resources. Meanwhile, seasonal changes significantly affect the nutrient composition and DM biomass of grasslands, and the lignification of forage hindered the digestion of livestock at the late stage of growth, presenting the dilemma of “strong in summer, fat in autumn, lean in winter and stuck in spring” (Yin et al. 2014; Zhang and Fu 2021). The energy requirements for livestock to survive also had a large difference in the cold and warm seasons in the alpine regions (Hynes et al. 2016; Saro et al. 2020). Therefore, from the perspective of seasonal variation, this study combines seasonal changes in forage nutrition value and livestock nutrient requirements to assess grassland carrying capacity.

The YRSP of the Tibetan Plateau in China is part of an important ecological security barrier and natural pasture, located in the Three‐River‐Source Parks (Sheng et al. 2019). The natural ecosystem of this region is typical and sensitive, and its biodiversity protection has an important position in the world (Lin and Zhao 2022). The main vegetation type is grassland, including alpine grassland and alpine meadow. This area has abundant wildlife resources and mainly depends on the grassland ecosystem for survival (Zhao et al. 2020). In recent years, with increasing conservation efforts, grassland carrying capacity has received widespread attention. Increasing numbers of grazing livestock and wildlife and overlapping habitats are putting further pressure on the grasslands and are one of the main constraints to sustainable development in the area (Cai et al. 2022; Gao et al. 2020; Ren et al. 2021). Calculating the number of herbivores that can be carried is therefore important for managing grassland use, protecting local wildlife, and managing grassland–livestock conflicts.

The aim of this study was to estimate interannual variation in carrying capacity per unit area by calculating annual and seasonal carrying capacity in two typical grassland types in YRSP combining the AGB of grasses, pasture nutrient status, and nutrient requirements of livestock. Additionally, by combining the two types of grassland areas, the seasonally and annually estimated number of standard SUs that can be carried by the two types of grassland was calculated. This assessment could provide a database for overstocking or understocking livestock in the region. Here we also calculate the grassland carrying pressure by comparing the calculation with the actual livestock number and wildlife number to further determine the grassland use status. The results provide support for the optimization of the regional herbivore feeding system and the rational allocation of resource space. We therefore attempted to address the following questions: (1) how do the seasonal nutrient dynamics vary in YRSP? (2) How does the grassland carrying capacity and seasonal dynamics types vary in the study area? (3) What is the status of grassland carrying pressure and what are the limiting factors of grassland carrying capacity?

## 2 Materials and Methods

### 2.1 Study Site

The YRSP is located in the southeast of the Qinghai–Tibet Plateau and the south of Qinghai Province. The geographical coordinates are 33°55′5″ ~ 35°28′15″ N and 97°1′20″ ~ 99°14′57″ E. The total area is 1.91 × 10^4^ km^2^, and the average altitude is over 4400 m. The region's climate is typical of continental plateaus. The average annual temperature is −4°C, the average annual precipitation is 200 ~ 500 mm, and the annual evaporation is approximately 1374.6 mm. YRSP is included in Maduo County, accounting for 78% of the county area. The region includes 19 administrative villages in Huanghe Township, Zhaling Township, and Machali Town. The vegetation types of Maduo County are main alpine meadow and alpine grassland. *Kobresia humilis*, *Kobresia pygmaea*, *Kobresia tibetica*, and *Koeleria cristatata* are the dominant species in the alpine meadow; and the dominant species in alpine steppe are *Stipa purpurea*, *Elymus nutans*, and *Poa crymophila* (Shu et al. 2022). It also has abundant wildlife resources, such as Tibetan wild ass (*Equus kuang*), Tibetan gazelle ( *Procapra picticaudata* ), and Tibetan fox ( *Vulpes ferrilata* ) (Ma et al. 2022).

### 2.2 Experimental Design and Sampling Point Survey

**FIGURE 1:**
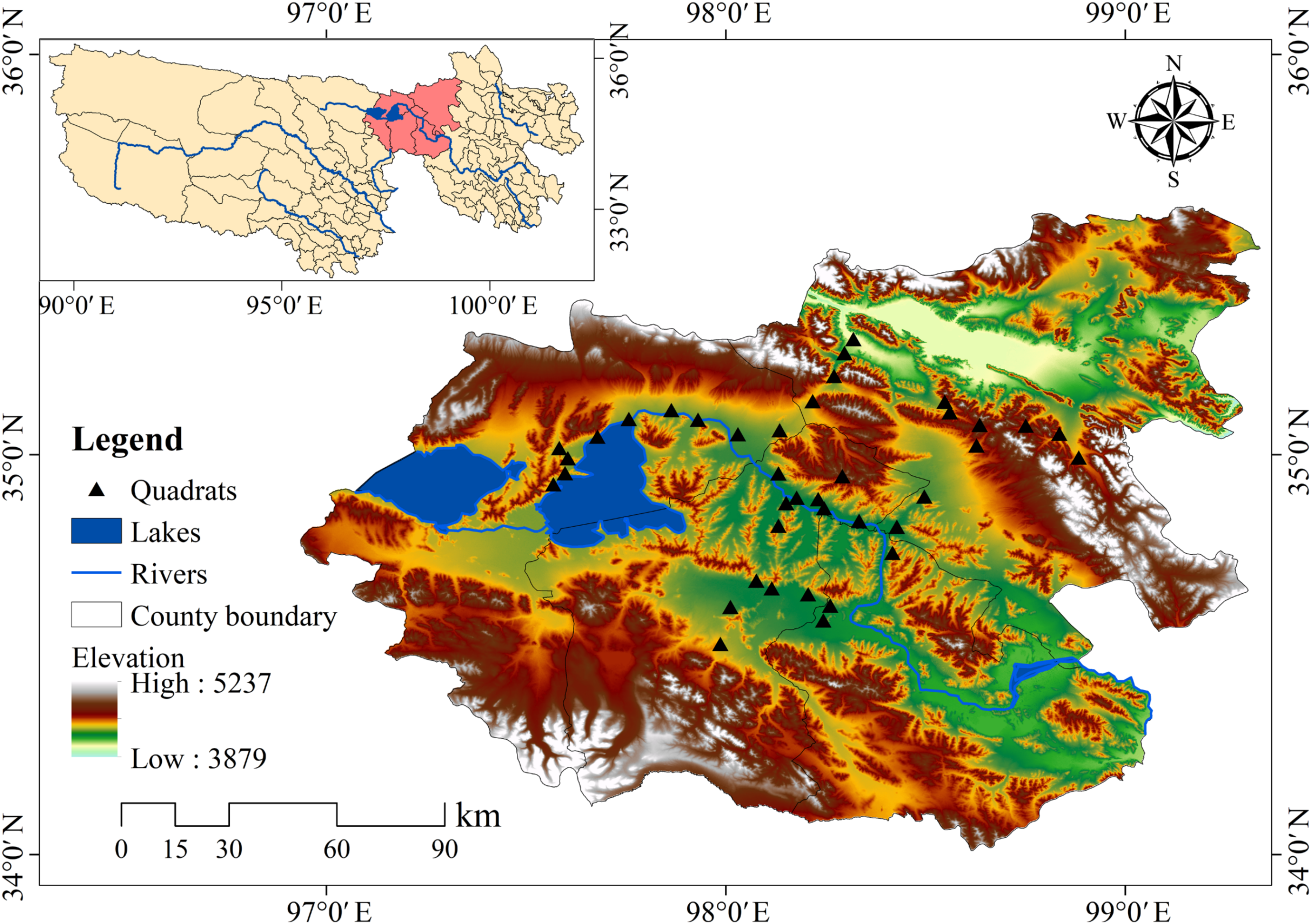
The spatial location and the sampling point of the study area.

Based on the geographical distribution characteristics of the YRSP, we conducted a natural grassland sample survey in Maduo County, and the survey route covered three townships in Maduo County, including Huanghe Township, Zhaling Township, and Machali Town (Figure 1). The quadrat survey was conducted along the main roads of the three townships, with each sample site approximately 10 km apart. Sample plots were selected 30 m away from the roadside to avoid the impact of the road on the survey. Four times surveys were investigated in flourishing period (August 2020), yellowing period (October 2020), wintering period (January 2021), and greening period (May 2021), respectively. The sampling point survey method was used with the OvitalMap application to identify longitude and latitude and to ensure the accuracy of each time survey. After removing the poorly recorded sample points, the sample points for the four seasonal surveys were 36, 33, 34, and 26 in August, October, January, and May, respectively. In the greening period survey, heavy snow caused some sample sites to be inaccessible.

At each sample site, the grassland type was determined according to the grassland dominant species and recorded the coordinates, elevation, soil texture, and grass type. The sample plots were surveyed using a random sampling method with five replicate samples taken from each sample point, and a total of 645 samples were taken. The size of each sample plot was 50 cm × 50 cm. The vegetation in the sample plots was harvested at ground level, removing inedible portions. The remaining material was then placed in separate envelopes and transported to the laboratory for further processing.

### 2.3 Determination of Forage Nutrients and Calculation of Carrying Capacity Per Unit Area

Considering both animal nutritional requirements and grass yield, three carrying capacities were calculated in this study to assess grassland carrying capacity, including dry matter carrying capacity (DMCC), digestible crude protein carrying capacity (DCPCC), and metabolic energy carrying capacity (MECC).

For this purpose, the collected forage samples were examined for multiple indicators, including AGB, crude protein (CP), ether extract (EE), neutral detergent fiber (NDF), acid detergent fiber (ADF), crude ash (Ash), calcium (Ca), and phosphorus (P) content of forages. Among them, the AGB, CP, and EE contents were used to calculate three grassland carrying capacities, and other indicators were used to evaluate the nutrient status of the grasslands in different grazing periods.

The collected forage samples were dried in an oven at 65°C to obtain AGB. According to the GB/T 6435‐2014, the forage DM was calculated directly by weighing after drying at 105°C for 24 h. After recording, the samples were crushed to pass through a 0.1 mm sieve for measurement. The CP content was determined by the Kjeldahl nitrogen determination method. The sample was boiled at 420°C for 1 h with concentrated sulfuric acid and copper sulfate. After that, the ammonia in the sample was distilled with a semiautomatic Kjeldahl nitrogen analyzer, and the CP was calculated by titrating and absorbing it with a hydrochloric acid standard solution. The EE was extracted by the Soxhlet extractor method. ADF and NDF contents were determined by the Van Soest method using an ANKOM A2000i fiber analyzer. The Ash content was estimated by placing dried samples in a muffle furnace at 500°C for 4 h and weighing residual Ash. The Ca and P concentrations were analyzed by inductively coupled plasma emission spectrometry (Donati, Amais, and Williams 2017). The ME was calculated using the following formula (Sun et al. 2015):

(1) ME=0.1456×GP+0.07675%×CP+0.1642%×EE+1.198

where GP (Gas production) is 24 h gas production (mL/200 mg feed), the flourishing and greening period is 46.50 mL, and the yellowing and wintering period is 39.67 mL.

According to forage nutrients and dry matter, we calculated three carrying capacities in different periods. Given the low carrying capacity during yellowing and wintering periods, we combined these into a single period and calculated carrying capacity for three distinct seasons: flourishing, greening, and withering. Three carrying capacities were estimated as follows:

(2) $$C_{\mathrm{DM}}=\frac{\mathrm{DM}\;\left(\mathrm{kg}\;{\mathrm{km}}^{-2}\right)\times \mathrm{UR}\left(\%\right)}{D\times \text{Daily intake of}\;\mathrm{dry}\;\text{matter}\;\left(\mathrm{kg}\;{\mathrm{km}}^{-2}\right)}$$

(3) $$C_{\mathrm{DCP}}=\frac{\mathrm{DCP}\;\left(\mathrm{kg}\;{\mathrm{km}}^{-2}\right)\times \mathrm{UR}\;\left(\%\right)}{D\times \text{Daily requirement of}\;\mathrm{DCP}\;\left(\mathrm{kg}\;{\mathrm{km}}^{-2}\right)}$$

(4) $$C_{\mathrm{ME}}=\frac{\mathrm{ME}\;\left(\mathrm{MJ}\;{\mathrm{km}}^{-2}\right)\times \mathrm{UR}\;\left(\%\right)}{D\times \text{Daily requirement of}\;\mathrm{ME}\;\left(\mathrm{kg}\;{\mathrm{km}}^{-2}\right)}$$

where UR is the utilization rate of the pasture. The UR is 30% in the greening period and 45% in the other three periods. *D* is the time of grazing in each period. Combining the grazing habits, interviews with grazers, and the results of He (He et al. 2021), we confirmed that the duration in the greening period was 49 days, the flourishing period was 113 days and the withering period was 203 days. According to the NY/T 635‐2015, the daily DM intake of a standard SU is 1.548 kg. The daily requirement of DCP and ME is 0.094 and 8.79 MJ days^−1^, respectively, to maintain weight of 45 kg (NY/T 635‐2015 2015). The daily requirements of DCP and ME are 0.1520 and 13.4 MJdays^−1^ when the standard SU increases by 100 g per day, which are 0.1920 and 16.8 MJ days^−1^ when the standard SU increases by 200 g per day. In accordance with actual production, we calculated the carrying capacity in each season based on an increase of 200 g of body weight per day during the flourishing season, 100 g of body weight per day during the greening season, and a constant weight during the withering season.

### 2.4 Calculation of the Carrying Number of Standard SU and GPI in Different Seasons

The carrying capacity of the standard SU is represented by the number of livestock being raised. The formula used was as follows:

(5) $$C_{{\mathrm{SU}}_{i}}=C_{i}\times A\;\left({\mathrm{km}}^{2}\right)\times \mathrm{UR}\;\left(\%\right)$$

The $C_{{\mathrm{SU}}_{i}}$ is the carrying number of standard SU in different seasons. $C_{i}$ denotes the carrying capacity per unit area of DM, DCP, and ME in three seasons. A represents the area of two grassland types. According to the data provided by the Bureau of Agriculture and Animal Husbandry and Forestry Science and Technology of Maduo Country, the livestock balance area was 5788.23 km^2^ and accounted for 25.68% of the total grassland area. The study of Yu et al. (2021) showed that alpine meadow and alpine grassland were the main types of grassland in Maduo Country, accounting for 76.66% and 23.34%, respectively.

The GPI is calculated by the following formula:

(6) $${\mathrm{GPI}}_{i}=\frac{C_{\text{Actual}}}{C_{{\mathrm{SU}}_{i}}}$$

(7) $${\mathrm{GPI}}_{\text{annual}}=\sum_{i}{\mathrm{GPI}}_{i}$$

In this formula, ${\mathrm{GPI}}_{i}$ is the grassland pressure index in i season (i represents the flourishing period, greening period, and withering period). ${\mathrm{GPI}}_{\text{annual}}$ is the annual grassland pressure index. The ${\mathrm{GPI}}_{i}$ and ${\mathrm{GPI}}_{\text{annual}}$ > 1, the number of large herbivores exceeds the optimum state, whereas for ${\mathrm{GPI}}_{i}$ and ${\mathrm{GPI}}_{\text{annual}}$ < 1, the grassland has the capacity to support more large herbivores. The detailed classification levels are shown in Table 1 (Zhao et al. 2024). The $C_{\text{Actual}}$ is the actual number of livestock in standard SUs. In Yu et al. (2021) study, the livestock number was 33.84 × 10^4^ SU and the wild ungulates were 8.24 × 10^4^ SU.

**TABLE 1:** Grassland pressure index classification levels.

Level	Grassland pressure index (*X*)
Affluent	*X* ≤ 0.5
Surplus	0.5 < *X* ≤ 0.9
Balance	0.9 < *X* ≤ 1.1
Critical overload	1.1 < *X* ≤ 1.3
Overload	*X* > 1.3

### 2.5 Data Analysis

Data were collated using Excel 2010 and analyzed using SPSS 22.0. *T*‐test was used to analyze forage biomass and nutritional data, metabolic energy, and carrying capacity of two grassland types. One‐way analysis (ANOVA) and Duncan's method were employed to investigate the four grazing seasons of forage nutritional data, metabolic energy, and carrying capacity. All data used *p* < 0.05 for a significant difference.

## 3 Results

### 3.1 AGB in Different Grassland Types and Seasons

**FIGURE 2:**
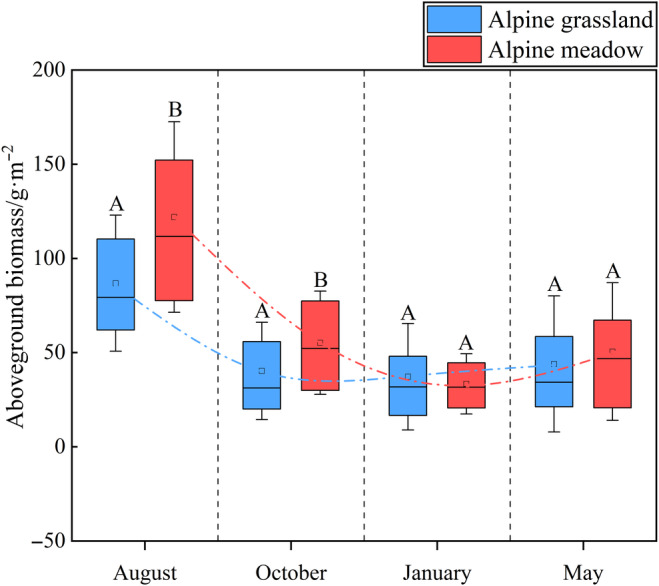
Aboveground biomass in four seasons and two grassland types. Capital letters mean significant differences at the 0.05 level.

During the season, AGB peaked in August in two grassland types, decreased in October and January, and increased in May (Figure 2). The AGB of alpine grassland in August, October, January, and May was 86.84, 40.29, 37.19, and 43.98 g m^−2^, respectively. And the AGB of alpine meadow was 121.96, 55.23, 33.40, and 50.56 g m^−2^, respectively. The alpine grassland had a lower AGB than the alpine meadow in August and October (*p* < 0.05), while in January and May, there were no significant differences between the two grassland types (*p* > 0.05).

### 3.2 Forage Nutrient Content in Different Grassland Types and Seasons

**FIGURE 3:**
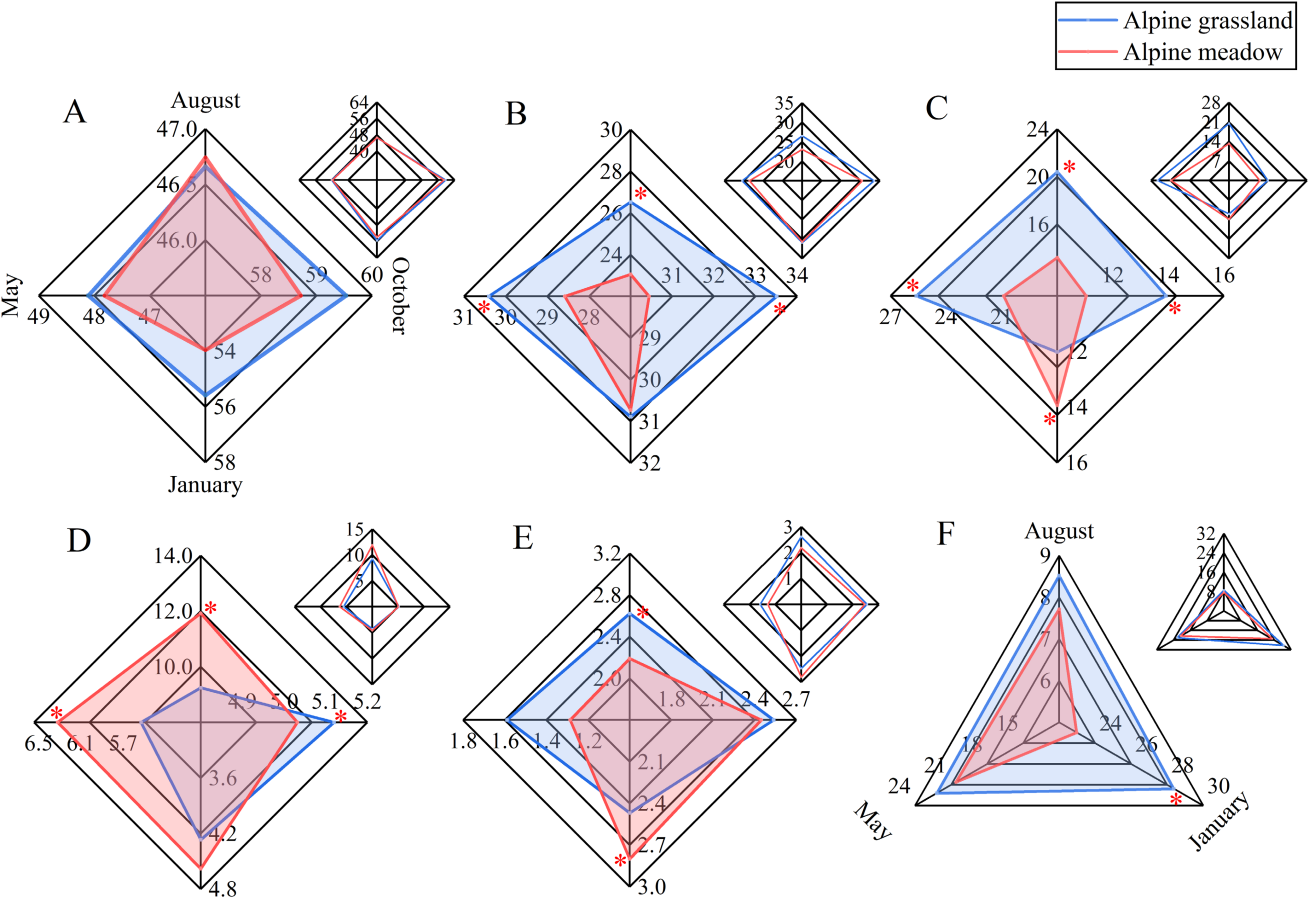
The response of forage nutrient content in different grassland types and seasons. A: NDF, B: ADF, C: Ash, D: CP, E: EE, F: Ca/P ratio. * indicates significant differences at the 0.05 level.

The nutrient content of forage was influenced by grassland type and season (Figure 3). Both NDF and ADF showed a similar change trend, with higher content in October and January, and lower content in August and May (*p* < 0.05). While Ash showed an opposite trend, with greater content in August and May, and lower content in October and January (*p* < 0.05). There was no significant difference in NDF content between the two grassland types across all four seasons. The ADF content in alpine grassland was higher than in alpine meadow in May, August, and October (*p* < 0.05). In alpine grassland, the Ash content was significantly higher than in alpine meadow in August, October, and May, but significantly lower in January.

The study found that the lowest CP content occurred in January, while the highest was observed in August. In August and May, the alpine meadow had a greater CP content compared to the alpine grassland, whereas in October, the alpine grassland had a higher CP content than the alpine meadow. The EE content was found to be the lowest in May compared to the other three seasons. Additionally, the EE content in the alpine meadow was significantly lesser than that in the alpine grassland in August, while greater in January. The Ca/P ratio was lost in January, so we only compared the other three seasons. The Ca/P ratio significantly changed as the seasons changed. The lowest Ca/P ratio was in August, and the highest Ca/P ratio was in January. The Ca/P ratio in alpine grassland was always greater than in alpine meadow in all three seasons, and there was a significant difference in January.

### 3.3 ME Content in Different Grassland Types and Seasons

The ME was calculated by formula 1. The ME exhibited a trend of being higher in August and May, and lower in October and January (Table 2). The order of ME content was January < October < May < August in two grassland types. Additionally, the ME content in alpine meadow was significantly greater than that in alpine grassland in August, October, and January (*p* < 0.05), while no significant difference was observed in May.

**TABLE 2:** ME content in different grassland types and seasons.

Indicator	Alpine grassland				Alpine meadow			
	August	October	January	May	August	October	January	May
ME (MJ)	7.979 ± 0.0 Aa	7.008 ± 0.0 Ba	6.982 ± 0.0 Ba	7.977 ± 0.0 Aa	7.981 ± 0.0 Ab	7.018 ± 0.0 Bb	6.982 ± 0.0 Bb	7.977 ± 0.0 Aa

*Note:* Different lowercase letters indicate that the indexes are significantly different under different grassland types in the same season (*p* < 0.05). Different capital letters indicate that the indexes are significantly different under different seasons in the same grassland types (*p* < 0.05). All values are expressed as the mean ± standard error. The same as below.

Abbreviation: ME, metabolic energy.

### 3.4 Nutrient Output in Different Grassland Types and Seasons

The results indicate that nutrient output in August was significantly higher than in the other three seasons (*p* < 0.05), which suggests that the flourishing period is the main nutrient supply period for grassland (Table 3). In August, the NDF, ADF, CP, DM, and ME were significantly lower in alpine grassland than in alpine meadow (*p* < 0.05), while the EE and Ash showed no significant difference between the two grassland types (*p* > 0.05). In October, the NDF, CP, EE, DM, and ME output in alpine grassland were significantly lower than in alpine meadow (*p* < 0.05). The NDF and Ash had no significant difference in the two grassland types in October (*p* > 0.05). In January and May, all indexes showed no significant difference between the two grassland types.

**TABLE 3:** Nutrient output in different grassland types and seasons.

Grassland types	Season	NDF, ×10^3^ kg km^−2^	ADF, ×10^3^ kg km^−2^	CP, ×10^3^ kg km^−2^	EE, ×10^3^ kg km^−2^	Ash, ×10^3^ kg km^−2^	DM ×10^3^ kg km^−2^	ME, ×10^3^ MJ km^−2^
Alpine grassland	August	40.22 ± 2.26 Ba	22.92 ± 1.25 Ba	7.81 ± 0.37 Ca	2.21 ± 0.13 Ba	16.77 ± 0.96 Ca	84.90 ± 3.83 Ba	676.70 ± 32.49 Ba
	October	23.65 ± 1.52 Aa	13.34 ± 0.85 Aa	2.17 ± 0.18 ABa	1.07 ± 0.08 Aa	5.14 ± 0.33 Aa	38.65 ± 2.61 Aa	282.95 ± 18.74 Aa
	January	19.84 ± 1.62 Aa	11.08 ± 0.90 Aa	1.59 ± 0.14 Aa	0.99 ± 0.08 Aa	4.30 ± 0.36 Aa	34.99 ± 2.84 Aa	254.24 ± 20.28 Aa
	May	19.22 ± 1.36 Aa	12.33 ± 0.93 Aa	2.38 ± 0.20 Ba	0.98 ± 0.10 Aa	12.55 ± 1.85 Ba	42.38 ± 3.90 Aa	353.03 ± 32.50 Aa
Alpine meadow	August	61.96 ± 4.88 Cb	30.01 ± 2.22 Cb	15.49 ± 1.07 Bb	2.72 ± 0.15 Ca	15.44 ± 1.06 Ca	126.51 ± 8.79 Cb	1041.32 ± 72.24 Bb
	October	32.25 ± 2.51 Bb	16.61 ± 1.28 Ba	2.88 ± 0.26 Ab	1.34 ± 0.12 Bb	5.98 ± 0.57 Aa	52.75 ± 4.17 Bb	388.20 ± 30.65 Ab
	January	17.42 ± 1.13 Aa	10.06 ± 0.71 Aa	1.57 ± 0.16 Aa	0.97 ± 0.08 Aa	4.75 ± 0.53 Aa	31.53 ± 2.13 Aa	227.48 ± 15.26 Aa
	May	23.64 ± 2.81 ABa	14.27 ± 1.72 ABa	3.11 ± 0.37 Aa	0.91 ± 0.11 Aa	9.26 ± 1.04 Ba	48.05 ± 5.63 ABa	403.23 ± 46.11 Aa

Abbreviations: ADF, acid detergent fiber; Ash, crude ash; CP, crude protein; DM, dry matter; EE, ether extract; ME, metabolic energy; NDF, neutral detergent fiber.

### 3.5 Carrying Capacity Per Unit Area in Different Grassland Types and Seasons

**FIGURE 4:**
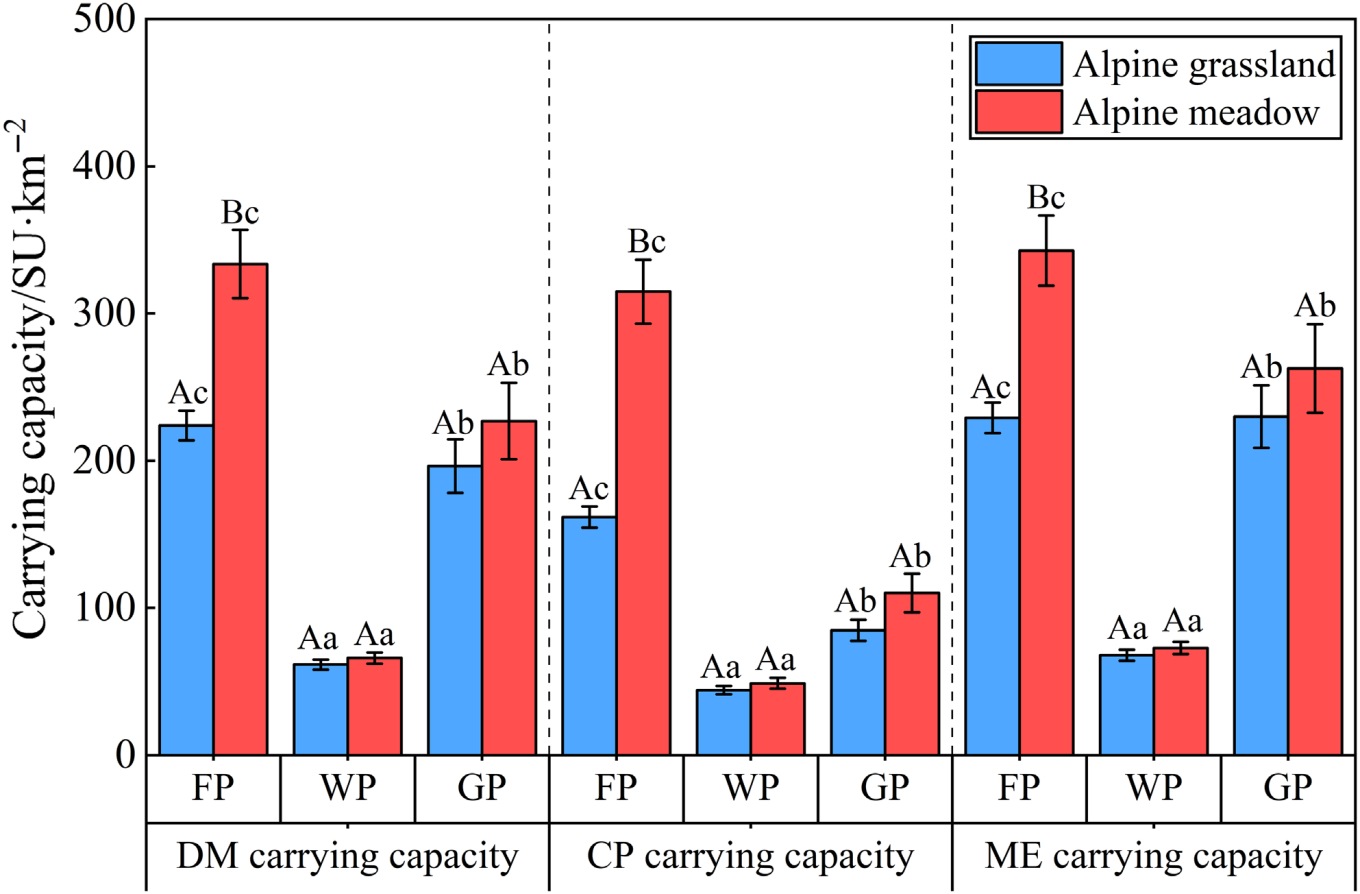
The change in three carrying capacities in different grassland types and grazing periods. Different capital letters indicate that each carrying capacity is significantly different under different grassland types in the same grazing period (*p* < 0.05). Different lowercase letters indicate that each carrying capacity is significantly different under different grazing periods in the same grassland type (*p* < 0.05). FP, flourishing period; GP, greening period; WP, withering period.

The ME carrying capacity was the greatest compared to the other two carrying capacities in three periods, while CP carrying capacity was the lowest. The DM carrying capacity varied from 61.51 ± 3.41 to 333.65 ± 23.18 SU km^−2^ in the three periods, CP carrying capacity varied from 44.18 ± 2.86 to 314.81 ± 21.67 SU km^−2^, and ME carrying capacity varied from 67.90 ± 3.73 to 342.71 ± 23.78 SU km^−2^. This indicates that the grassland carrying capacity showed significant seasonality (Figure 4). On the seasonal scale, the three carrying capacities exhibited a similar trend, with the highest in the flourishing period and the lowest in the withering period. Compared with alpine grassland, the alpine meadow demonstrated a consistently high carrying capacity throughout all grazing periods, with a significant difference observed during the flourishing period (*p* < 0.05). In the withering and greening periods, there was no significant difference in carrying capacity between the two grassland types (*p* > 0.05).

### 3.6 The Number of Carrying Standard SU in Different Grassland Types and Seasons

The seasonal carrying number and annual carrying number of standard SU in two grassland types are shown in Table 4. In the DM carrying capacity, the seasonal carrying number in two grassland types was 77.89 × 10^4^, 14.53 × 10^4^, and 32.09 × 10^4^ SU in flourishing period, withering period, and greening period, respectively. The annual quantity was 36.50 × 10^4^ SU. In the CP carrying capacity, the seasonal carrying number in flourishing period, withering period, and greening period were 74.21 × 10^4^, 12.43 × 10^4^, and 30.90 × 10^4^ SU. The annual carrying number was 34.03 × 10^4^ SU. According to the ME carrying capacity calculation, the annual carrying number was 35.00 × 10^4^ SU, and the seasonal carrying number in two grassland types was 59.32 × 10^4^, 18.66 × 10^4^, and 46.59 × 10^4^ SU in flourishing period, withering period, and greening period.

**TABLE 4:** Seasonal carrying number and annual carrying number in different periods and grassland types.

Index	Grazing Period	Seasonal carrying quantity in alpine grassland (10^4^ SU)	Seasonal carrying quantity in alpine meadow (10^4^ SU)	Seasonal carrying quantity in two grassland types (10^4^ SU)	Annual carrying quantity (10^4^ SU)
DM carrying capacity	FP	12.91	64.98	77.89	36.50
	WP	3.21	11.32	14.53	
	GP	6.79	25.30	32.09	
CP carrying capacity	FP	10.04	64.17	74.21	34.04
	WP	2.69	9.74	12.43	
	GP	5.87	25.04	30.90	
ME carrying capacity	FP	10.03	49.29	59.32	35.00
	WP	4.13	14.53	18.66	
	GP	9.81	36.79	46.59	

### 3.7 Grassland Carrying Pressure in Different Grassland Types and Seasons

**FIGURE 5:**
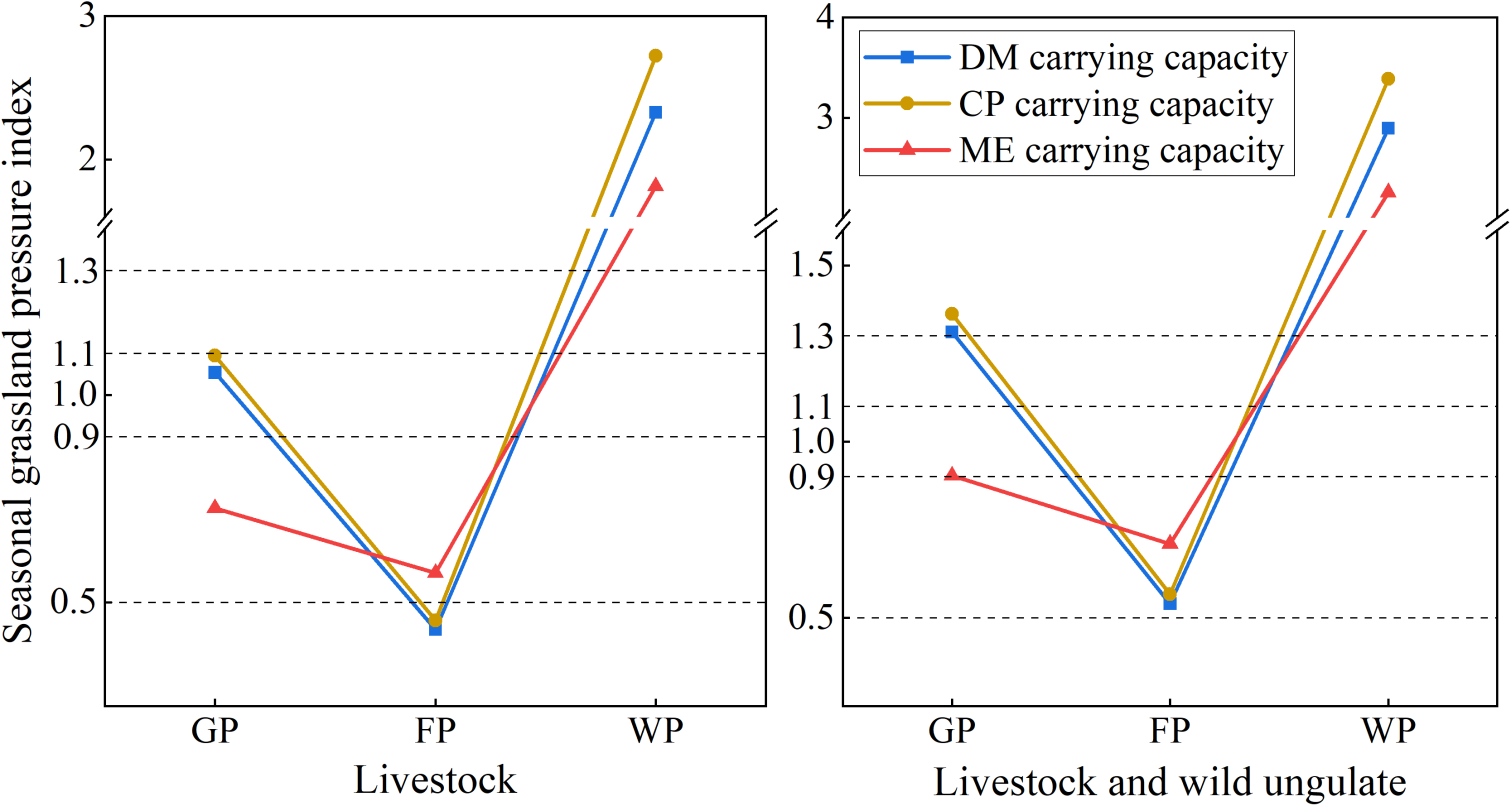
Comparison of seasonal grassland carrying pressure in the YRSP.

The GPI was calculated with two scenarios (Figure 5). One considers only the ratio of the actual number of livestock to the theoretical number, and the other considers the ratio of the actual number of livestock to the combined number of livestock and wild ungulate species. Based on the GPI classification level, the GPI of DM, CP, and ME carrying capacity in the flourishing period were < 0.9, where the DM and CP carrying capacity only considered livestock were < 0.5, indicating that the grassland was in surplus in this period. In the withering period, the GPI varies from 1.81 to 3.39 (GPI > 1.3), indicating that the grassland was overloaded during this period. During the greening period, when livestock number was only considered, GPI was in balance in DM and CP carrying capacity (0.9 < GPI < 1.1) and in surplus in the ME carrying capacity (0.5 < GPI < 0.9). The GPI of DM and CP carrying capacity were overloaded (GPI > 1.3) and GPI of ME carrying capacity was in balance when considering the number of livestock and wild ungulates (0.9 < GPI < 1.1).

**FIGURE 6:**
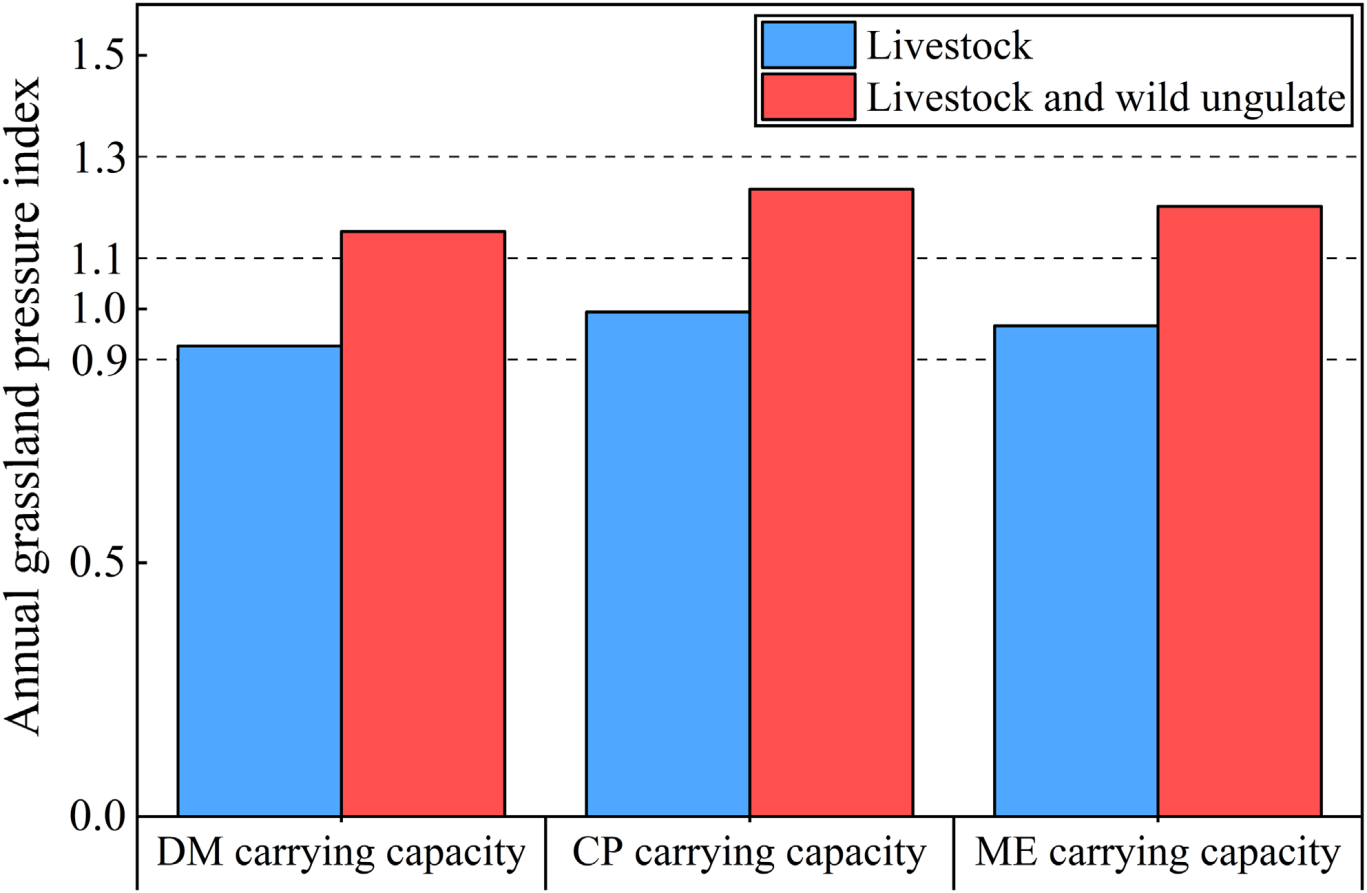
Comparison of annual grassland pressure index in the YRSP.

The annual GPI associated with three carrying capacities showed that the grassland was critically overloaded (1.1 < GPI < 1.3) when wild ungulates and livestock were taken into account, and balanced when wildlife was excluded (0.9 < GPI < 1.1). The annual GPI showed a trend of DM carrying capacity < ME carrying capacity < CP carrying capacity (Figure 6).

## 4 Discussion

Maduo County is situated within the YRSPs and is regarded as an ecological security defense line in the Three‐River‐Source Parks Ecological Protection and Construction project (Fan et al. 2010). With the promotion of conservation, the theoretical carrying capacity of grassland has greatly improved. However, the high livestock population and the overlapping niches of wildlife and livestock still is the main reasons for grassland overload on this site (Shu et al. 2022). Some studies have indicated that disregarding wildlife populations may result in an underestimation of the GPI, which in turn can lead to the overloading of grasslands (Ren et al. 2021; Zhao et al. 2020). Existing studies also have tended to focus on annual carrying capacity and less on seasonal variations in carrying capacity (De Leeuw et al. 2019; Fang et al. 2021; F. Yang et al. 2018). Therefore, this study calculates the grassland carrying capacity and pressure index by investigating the biomass and nutritive value of grassland in different seasons to provide theoretical guidance for the rational use of grassland and ecological stability protection.

### 4.1 Seasonal Characteristics of Above‐Ground Biomass and Nutrient Content

AGB and nutritive value of pasture grasses are affected by seasonal variation and grassland type. Previous studies have demonstrated that herbage growth is rapid during the greening and flourishing periods, accompanied by the accumulation of nutrients and biomass (Liu et al. 2021; Zaret et al. 2022). However, as the growing season ends, the rate of forage growth slows or even stops, and the nutrient composition and biomass decrease (Zha et al. 2022). In this study, the AGB and forage nutrient content were consistent with previous changes (Dai et al. 2020). The range of biomass variation was 33.40 ~ 121.96 g m^−2^, which was lower than the yield at low altitudes. This was mainly attributed to the characteristics of high altitude, harsh natural conditions, and short growing seasons in the study area (He et al. 2021). The variation range of CP content was found to be 4.3% to 11.92%, with the ADF and NDF varying from 23.06% to 34.08% and 44.79% to 60.16%, respectively. The CP content of forage ≥ 16% was considered superior, 10% ~ 15% was considered medium, and ≤ 10% was inferior (Saro et al. 2020). The standard for high‐quality forage was ADF lower than 33% (Du et al. 2023). In this study, the CP content of overall pasture grasses was in the lower middle class, while the ADF was in the higher level, indicating that pasture grasses in this region have low CP content and should be supplemented with some protein in feeding.

### 4.2 Seasonal Characteristics of Nutrient Production, Carrying Capacity, and Carrying Numbers

Nutrient output per unit area is a key determinant of the grassland carrying capacity of herbivores. Our results indicate that the nutrient output decreased after August and increased in May. In August, it was significantly higher than in the other seasons, even higher than the combined output of October and January. This suggests that there is an imbalance in nutrient output between the different seasons. Similarly, the carrying capacity per unit area also exhibits significant seasonal fluctuations. The results of this study showed that the carrying capacity per unit area was the lowest during the withering period and highest during the flourishing period, which was about three times that of the withering period. The CP carrying capacity per unit area was found to be the smallest among the three carrying capacities, which differed from the results of previous studies (Cai et al. 2022; Sun et al. 2020). This may be attributed to the calculation method employed. The ME carrying capacity per unit area was the largest among the three carrying capacities. This may be due to the significant decrease in CP content observed during the winter and spring months, while the EE content remained at a high level (Gao et al. 2020). Our results also showed that alpine meadow had higher nutrient output and carrying capacity than alpine grassland. This may be related to the characteristics of the two grassland types, which was consistent with the findings of Dai et al. (Dai et al. 2020). Thus, it is necessary to distinguish between the utilization efficiency of the two grasslands in order to avoid overload or surplus.

The calculation of the annual livestock carrying numbers showed that the DM, CP, and ME carrying numbers are 36.50 × 10^4^, 34.04 × 10^4^, and 35.00 × 10^4^ SU, all greater than the actual livestock (33.84 × 10^4^ SU) (Yu et al. 2021). The DM, CP, and ME carrying numbers in the flourishing period were 77.89 × 10^4^, 74.21 × 10^4^, and 59.32 × 10^4^ SU, respectively. And the carrying number in DM, CP, and ME at the withering period were 14.53 × 10^4^, 12.43 × 10^4^, and 18.66 × 10^4^ SU, respectively. This evidence indicates that under the current grazing allocation system, the grassland used in the YRSP as a whole is in equilibrium. However, the grassland is underutilized during the flourishing period and overgrazed during the withering period.

### 4.3 Assessment of the GPI and Recommendations for Adaptive Management

GPI reflects the pressure state of grassland and represents the ability of the grassland ecosystem to undertake risks. When the GPI exceeds the warning value, effective measures should be taken to alleviate the grassland pressure and avoid the ecological risk caused by the high GPI. Previous studies also have shown that not considering wildlife populations can underestimate grassland carrying pressure and lead to grassland overload (Cai et al. 2022). Thus the GPI was calculated by two aspects and it was found that in general, the grassland in YRSP was in equilibrium when wildlife was not considered, while the carrying capacity of grassland was in critical overload when both wildlife and livestock were considered, which is similar to the results of Ren et al. (2021). The GPI was in the surplus level (GPI < 0.9) in the flourishing period, and DM carrying capacity, as a more easily quantifiable and calculated indicator, could be utilized as an indicator to measure the grassland carrying capacity during this season. In contrast, during the withering period, CP content was the limiting factor, and CP carrying capacity should be considered first in order to comply with the goal of ecological conservation. During the greening period, the GPI was the critical overload level (1.1 < GPI ≤ 1.3) when both livestock and wildlife were considered and the equilibrium level (0.9 < GPI ≤ 1.1) when wildlife was not considered. However, as forage was fragile during the greening period, grazing could significantly slow down forage germination and reduce photosynthesis, which could affect subsequent grass growth and yield (Liu 2022). Therefore, the carrying capacity during the regrowth period should be less than the calculated minimum value (CP carrying capacity).

The GPI is far exceeded the overloaded grazing level (GPI > 1.3) in the withering period. At this time, the diet of livestock and wild animals can no longer be solved by grazing alone. According to the minimum carrying number (CP carrying number) in this season, removing the number of wild animals, only 4.19 × 10^4^ SU standard sample units were carried. The geographical location and ecological protection status of the YRSP should give priority to ensuring wildlife herbivory. Therefore, the implementation of cold‐season supplementary feeding and increasing the amount of livestock sales at the end of flourishing period are important ways to reduce grazing pressure and protect the sustainable utilization of grassland (Xu, Quan, et al. 2020; Xu, Wang, et al. 2020; Zhang et al. 2014, 2019). Based on the calculations in this article, at least 426.50 t of forage should be supplemented to domestic livestock to ensure adequate grass intake for herbivores.

This study aims to protect the natural grasslands in the YRSP and provides a reference for the rational use of grasslands to achieve a balance of demand between wildlife and livestock, as well as the formulation of reasonable conservation and management policies. It is worth noting that our study still has some limitations. First, due to interannual climate and global warming trends, grassland production, and nutrients in the YRSP have some ups and downs, so long‐term monitoring should be implemented to avoid overgrazing caused by interannual variations. Second, although the GPI of wildlife and domestic animals was calculated from two aspects in this study, long‐term surveys and monitoring of their populations are still needed due to the migratory nature of wildlife. Therefore, future studies should pay attention to these issues to understand and protect grasslands more comprehensively.

## 5 Conclusion

This study took the typical alpine grassland and alpine meadow in the YRSP as the research object and analyzed the grassland carrying capacity and GPI in different seasons through the investigation and calculation of forage yield and nutrient composition in different grazing periods. It was found that the yield, nutritional characteristics, and unit area carrying capacity of the two types of grassland in YRSP had obvious seasonality and the general characteristics of different grazing periods were as follows: flourishing period > greening period > withering period, and these indexes of alpine meadows were higher than alpine grassland as a whole. The MECC was the highest and CP carrying capacity was the lowest among the three types of carrying capacity calculated. By calculating the GPI, it was found that the annual carrying capacity of the YRSP was in equilibrium when only the number of livestock was considered, while the annual carrying capacity was overloaded when the number of wildlife and livestock was considered. Overall, the grassland was at surplus level during the flourishing period, at a critical overload level during the greening period, and at an overloaded level during the withering period. Therefore, from the point of view of ecological protection, the easily accessible DM content of the forage could be used as grassland carrying capacity indicator in the flourishing period, and CP was the limiting factor in the withering period, which should be used as grassland carrying capacity indicator. It is recommended that the YRSP should be adequately supplemented or livestock numbers reduced during the withering and greening periods to ensure the survival and reproduction of wildlife and the rational use and sustainable development of the grasslands in the area.

## Author Contributions

**Yalin Wang:** visualization (equal), writing – original draft (equal). **Yuanyue Geng:** data curation (equal), investigation (equal). **Xungang Wang:** investigation (equal), methodology (equal). **Tianwei Xu:** conceptualization (equal), supervision (equal). **Qian Zhang:** investigation (equal). **Hongjin Liu:** formal analysis (equal), resources (equal). **Na Zhao:** data curation (equal), software (equal). **Shixiao Xu:** funding acquisition (equal), methodology (equal), project administration (equal).

## Conflicts of Interest

The authors declare no conflicts of interest.

## Supporting information


Data S1.

## Data Availability

The data that support the findings of this study are available in the Supporting Information of this article.

## References

[ece370586-bib-0002] Blair, J. , J. Nippert , and J. Briggs . 2014. “Grassland Ecology.” In Ecology and the Environment, edited by R. K. Monson , 389–423. New York, NY: Springer. 10.1007/978-1-4614-7501-9_14.

[ece370586-bib-0003] Cai, Z. , P. Song , J. Wang , et al. 2022. “Grazing Pressure Index Considering Both Wildlife and Livestock in Three‐River Headwaters, Qinghai–Tibetan Plateau.” Ecological Indicators 143: 109338. 10.1016/j.ecolind.2022.109338.

[ece370586-bib-0004] Cao, Y. , J. Wu , X. Zhang , et al. 2019. “Dynamic Forage‐Livestock Balance Analysis in Alpine Grasslands on the Northern Tibetan Plateau.” Journal of Environmental Management 238: 352–359. 10.1016/j.jenvman.2019.03.010. 30856595

[ece370586-bib-0006] Chapman, E. J. , and C. J. Byron . 2018. “The Flexible Application of Carrying Capacity in Ecology.” Global Ecology and Conservation 13: e00365. 10.1016/j.gecco.2017.e00365.

[ece370586-bib-0007] Dai, L. , X. Guo , X. Ke , et al. 2020. “Biomass Allocation and Productivity–Richness Relationship Across Four Grassland Types at the Qinghai Plateau.” Ecology and Evolution 10: 506–516. 10.1002/ece3.5920. 31988738 PMC6972799

[ece370586-bib-0008] De Leeuw, J. , A. Rizayeva , E. Namazov , et al. 2019. “Application of the MODIS MOD 17 Net Primary Production Product in Grassland Carrying Capacity Assessment.” International Journal of Applied Earth Observation and Geoinformation 78: 66–76. 10.1016/j.jag.2018.09.014.

[ece370586-bib-0009] Donati, G. L. , R. S. Amais , and C. B. Williams . 2017. “Recent Advances in Inductively Coupled Plasma Optical Emission Spectrometry.” Journal of Analytical Atomic Spectrometry 32: 1283–1296. 10.1039/C7JA00103G.

[ece370586-bib-0010] Du, Y. , X. Ke , L. Dai , G. Cao , H. Zhou , and X. Guo . 2020. “Moderate Grazing Increased Alpine Meadow Soils Bacterial Abundance and Diversity Index on the Tibetan Plateau.” Ecology and Evolution 10: 8681–8687. 10.1002/ece3.6563. 32884650 PMC7452759

[ece370586-bib-0011] Du, Y. , Y. Zhang , X. Chai , et al. 2023. “Effects of Different Tillage Systems and Mowing Time on Nutrient Accumulation and Forage Nutritive Value of *Cyperus esculentus* .” Frontiers in Plant Science 14: 1162572. 10.3389/fpls.2023.1162572. 37123851 PMC10140299

[ece370586-bib-0012] Fan, J. W. , Q. Q. Shao , J. Y. Liu , et al. 2010. “Assessment of Effects of Climate Change and Grazing Activity on Grassland Yield in the Three Rivers Headwaters Region of Qinghai–Tibet Plateau, China.” Environmental Monitoring and Assessment 170: 571–584. 10.1007/s10661-009-1258-1. 20041346

[ece370586-bib-0013] Fang, Y. , F. Zhu , S. Yi , X. Qiu , and Y. Ding . 2021. “Ecological Carrying Capacity of Alpine Grassland in the Qinghai–Tibet Plateau Based on the Structural Dynamics Method.” Environment, Development and Sustainability 23: 12550–12578. 10.1007/s10668-020-01182-2.

[ece370586-bib-0014] Fetzel, T. , P. Havlik , M. Herrero , and K. H. Erb . 2017. “Seasonality Constraints to Livestock Grazing Intensity.” Global Change Biology 23: 1636–1647. 10.1111/gcb.13591. 27976453

[ece370586-bib-0015] Freeland, W. J. , and D. Choquenot . 1990. “Determinants of Herbivore Carrying Capacity: Plants, Nutrients, and *Equus Asinus* in Northern Australia.” Ecology 71: 589–597. 10.2307/1940312.

[ece370586-bib-0016] Gao, H. , F. Jiang , X. Chi , et al. 2020. “The Carrying Pressure of Livestock Is Higher Than That of Large Wild Herbivores in Yellow River Source Area, China.” Ecological Modelling 431: 109163. 10.1016/j.ecolmodel.2020.109163.

[ece370586-bib-0017] He, F. , D. Chen , Q. Lin , et al. 2021. “Temporal and Spatial Patterns of Herbage and Nutrient Carrying Capacity of Alpine Grassland of Sanjiangyuan.” Acta Agrestia Sinica 29: 2808–2816. 10.11733/j.issn.1007-0435.2021.12.022.

[ece370586-bib-0018] Herrero, M. , P. Havlík , H. Valin , et al. 2013. “Biomass Use, Production, Feed Efficiencies, and Greenhouse Gas Emissions From Global Livestock Systems.” Proceedings of the National Academy of Sciences 110: 20888–20893. 10.1073/pnas.1308149110. PMC3876224 24344273

[ece370586-bib-0019] Herrero, M. , P. K. Thornton , P. Gerber , and R. S. Reid . 2009. “Livestock, Livelihoods and the Environment: Understanding the Trade‐Offs.” Current Opinion in Environmental Sustainability 1: 111–120. 10.1016/j.cosust.2009.10.003.

[ece370586-bib-0020] Hynes, D. N. , S. Stergiadis , A. Gordon , and T. Yan . 2016. “Effects of Crude Protein Level in Concentrate Supplements on Animal Performance and Nitrogen Utilization of Lactating Dairy Cows Fed Fresh‐Cut Perennial Grass.” Journal of Dairy Science 99: 8111–8120. 10.3168/jds.2016-11110. 27522417

[ece370586-bib-0022] Li, W. , C. Liu , W. Wang , et al. 2021. “Effects of Different Grazing Disturbances on the Plant Diversity and Ecological Functions of Alpine Grassland Ecosystem on the Qinghai–Tibetan Plateau.” Frontiers in Plant Science 12: 765070. 10.3389/fpls.2021.765070. 34966399 PMC8710682

[ece370586-bib-0023] Lin, H. , and Y. Zhao . 2022. “Soil Erosion Assessment of Alpine Grassland in the Source Park of the Yellow River on the Qinghai–Tibetan Plateau, China.” Frontiers in Ecology and Evolution 9: 771439. 10.3389/fevo.2021.771439.

[ece370586-bib-0052] Liu, Y. 2022. “Grazing Rest during Spring Regreening Period Promotes the Ecological Restoration of Degraded Alpine Meadow Vegetation through Enhanced Plant Photosynthesis and Respiration.” Frontiers in Plant Science 13. 10.3389/fpls.2022.1008550. PMC9574263 36262656

[ece370586-bib-0024] Liu, D. , C. Zhang , R. Ogaya , M. Fernández‐Martínez , T. A. M. Pugh , and J. Peñuelas . 2021. “Increasing Climatic Sensitivity of Global Grassland Vegetation Biomass and Species Diversity Correlates With Water Availability.” New Phytologist 230: 1761–1771. 10.1111/nph.17269. 33577084 PMC8252445

[ece370586-bib-0025] Ma, B. , W. Zeng , Y. Xie , et al. 2022. “Boundary Delineation and Grading Functional Zoning of Sanjiangyuan National Park Based on Biodiversity Importance Evaluations.” Science of the Total Environment 825: 154068. 10.1016/j.scitotenv.2022.154068. 35217041

[ece370586-bib-0026] Meshesha, D. T. , M. Moahmmed , and D. Yosuf . 2019. “Estimating Carrying Capacity and Stocking Rates of Rangelands in Harshin District, Eastern Somali Region, Ethiopia.” Ecology and Evolution 9: 13309–13319. 10.1002/ece3.5786. 31871646 PMC6912879

[ece370586-bib-0027] NY/T 635‐2015 . 2015. “Agricultural Industry Standard of the People's Republic of China: Calculation of Rangeland Carrying Capacity. Ministry of Agriculture and Rural Affairs of the People's Republic of China.” (accessed on 1 November 2024). https://www.biaozhun.org/hangye/82311.html.

[ece370586-bib-0028] Piipponen, J. , M. Jalava , J. de Leeuw , et al. 2022. “Global Trends in Grassland Carrying Capacity and Relative Stocking Density of Livestock.” Global Change Biology 28: 3902–3919. 10.1111/gcb.16174. 35320616 PMC9321565

[ece370586-bib-0029] Ren, Y. , Y. Zhu , D. Baldan , et al. 2021. “Optimizing Livestock Carrying Capacity for Wild Ungulate‐Livestock Coexistence in a Qinghai–Tibet Plateau Grassland.” Scientific Reports 11: 3635. 10.1038/s41598-021-83207-y. 33574501 PMC7878488

[ece370586-bib-0030] Saro, C. , J. Mateo , I. Caro , et al. 2020. “Effect of Dietary Crude Protein on Animal Performance, Blood Biochemistry Profile, Ruminal Fermentation Parameters and Carcass and Meat Quality of Heavy Fattening Assaf Lambs.” Animals 10: 2177. 10.3390/ani10112177. 33233459 PMC7700360

[ece370586-bib-0031] Sheng, W. , L. Zhen , Y. Xiao , and Y. Hu . 2019. “Ecological and Socioeconomic Effects of Ecological Restoration in China's Three Rivers Source Region.” Science of the Total Environment 650: 2307–2313. 10.1016/j.scitotenv.2018.09.265. 30292990

[ece370586-bib-0032] Shu, K. , X. Gao , D. Qian , L. Zhao , Q. Li , and L. Dai . 2022. “Relationship Between Biomass and Biodiversity of Degraded Grassland in the Sanjiangyuan Region of Qinghai–Tibet Plateau.” Diversity 14: 1002. 10.3390/d14111002.

[ece370586-bib-0033] Sun, J. , P. Wang , Y. Tong , et al. 2020. “Overload Type and Optimization of Meadow Carrying Capacity in Maqin County in the Three‐River Source Region, China.” Journal of Mountain Science 17: 1387–1397. 10.1007/s11629-019-5904-y.

[ece370586-bib-0034] Sun, P. , Z. Cui , S. Liu , C. Chai , L. Hao , and X. Wang . 2015. “Seasonal Evaluation of Nutrition and Carrying Capacity of Grazing Pastures in the Three‐River Source Region.” Acta Prataculturae Sinica 24: 92–101. 10.11686/cyxb2015024.

[ece370586-bib-0035] Umuhoza, J. , G. Jiapaer , H. Yin , et al. 2021. “The Analysis of Grassland Carrying Capacity and Its Impact Factors in Typical Mountain Areas in Central Asia—A Case of Kyrgyzstan and Tajikistan.” Ecological Indicators 131: 108129. 10.1016/j.ecolind.2021.108129.

[ece370586-bib-0036] Xu, B. , X. C. Yang , W. G. Tao , et al. 2008. “MODIS‐Based Remote Sensing Monitoring of Grass Production in China.” International Journal of Remote Sensing 29: 5313–5327. 10.1080/01431160802036276.

[ece370586-bib-0037] Xu, T. , X. Quan , X. Zhang , et al. 2020. “Sustainable Development of Ecological Grass‐Based Livestock in Qinghai–Tibet Plateau Alpine Area: Principle, Technology and Practice.” Acta Ecologica Sinica 40: 6324–6337. 10.5846/stxb201912142705.

[ece370586-bib-0038] Xu, T. , X. Wang , X. Zhao , et al. 2020. “Standing Herbage Nutrition Characters and Herbivore Carrying Capacities of Typical Alpine Grasslands in Sanjiangyuan National Park During Cold Season.” Chinese Science Bulletin 65: 3610–3618. 10.1360/TB-2020-0118.

[ece370586-bib-0039] Yang, C. , P. Gao , F. Hou , et al. 2018. “Relationship Between Chemical Composition of Native Forage and Nutrient Digestibility by Tibetan Sheep on the Qinghai–Tibetan Plateau.” Journal of Animal Science 96: 1140–1149. 10.1093/jas/sky002. 29617805 PMC6140931

[ece370586-bib-0040] Yang, F. , Q. Shao , X. Guo , et al. 2018. “Effect of Large Wild Herbivore Populations on the Forage‐Livestock Balance in the Source Region of the Yellow River.” Sustainability 10: 340. 10.3390/su10020340.

[ece370586-bib-0041] Yang, Y. , D. Zhao , and H. Chen . 2022. “Full Title: Quantifying the Ecological Carrying Capacity of Alpine Grasslands on the Qinghai–Tibet Plateau.” Ecological Indicators 136: 108634. 10.1016/j.ecolind.2022.108634.

[ece370586-bib-0042] Yin, F. , X. Deng , Q. Jin , Y. Yuan , and C. Zhao . 2014. “The Impacts of Climate Change and Human Activities on Grassland Productivity in Qinghai Province, China.” Frontiers of Earth Science 8: 93–103. 10.1007/s11707-013-0390-y.

[ece370586-bib-0043] Yu, H. , B. Liu , G. Wang , et al. 2021. “Grass‐Livestock Balance Based Grassland Ecological Carrying Capability and Sustainable Strategy in the Yellow River Source National Park, Tibet Plateau, China.” Journal of Mountain Science 18: 2201–2211. 10.1007/s11629-020-6087-2.

[ece370586-bib-0044] Zaret, M. M. , M. A. Kuhs , J. C. Anderson , E. W. Seabloom , E. T. Borer , and L. L. Kinkel . 2022. “Seasonal Shifts From Plant Diversity to Consumer Control of Grassland Productivity.” Ecology Letters 25: 1215–1224. 10.1111/ele.13993. 35229976 PMC9544143

[ece370586-bib-0045] Zha, X. , Y. Tian , Ouzhu , and G. Fu . 2022. “Response of Forage Nutrient Storages to Grazing in Alpine Grasslands.” Frontiers in Plant Science 13: 991287. 10.3389/fpls.2022.991287. 36388576 PMC9664390

[ece370586-bib-0046] Zhang, H. , and G. Fu . 2021. “Responses of Plant, Soil Bacterial and Fungal Communities to Grazing Vary With Pasture Seasons and Grassland Types, Northern Tibet.” Land Degradation & Development 32: 1821–1832. 10.1002/ldr.3835.

[ece370586-bib-0047] Zhang, J. , L. Zhang , W. Liu , Y. Qi , and X. Wo . 2014. “Livestock‐Carrying Capacity and Overgrazing Status of Alpine Grassland in the Three‐River Headwaters Region, China.” Journal of Geographical Sciences 24: 303–312. 10.1007/s11442-014-1089-z.

[ece370586-bib-0048] Zhang, J. , L. Zhang , X. Liu , and Q. Qiao . 2019. “Research on Sustainable Development in an Alpine Pastoral Area Based on Equilibrium Analysis Between the Grassland Yield, Livestock Carrying Capacity, and Animal Husbandry Population.” Sustainability 11: 4659. 10.3390/su11174659.

[ece370586-bib-0049] Zhang, Z. , Y. Zhao , H. Lin , et al. 2023. “Comprehensive Analysis of Grazing Intensity Impacts Alpine Grasslands Across the Qinghai–Tibetan Plateau: A Meta‐Analysis.” Frontiers in Plant Science 13: 1083709. 10.3389/fpls.2022.1083709. 36733589 PMC9887153

[ece370586-bib-0050] Zhao, X. , T. Xu , J. Ellis , F. He , L. Hu , and Q. Li . 2020. “Rewilding the Wildlife in Sangjiangyuan National Park, Qinghai–Tibetan Plateau.” Ecosystem Health and Sustainability 6: 1776643. 10.1080/20964129.2020.1776643.

[ece370586-bib-0051] Zhao, Y. J. , W. J. Song , Z. S. Wu , et al. 2024. “Spatial and Temporal Analysis of Reasonable Livestock Carrying Capacity and Ecological Carrying Capacity of Grasslands Inpastoral Areas of China.” [In Chinese.] Chinese Science Bulletin: 1–16. 10.1360/TB-2024-0346.

